# Optimizing molluscicide treatment strategies in different control stages of schistosomiasis in the People’s Republic of China

**DOI:** 10.1186/1756-3305-5-260

**Published:** 2012-11-14

**Authors:** Guo-Jing Yang, Le-Ping Sun, Qing-Biao Hong, Hong-Ru Zhu, Kun Yang, Qi Gao, Xiao-Nong Zhou

**Affiliations:** 1 Jiangsu Institute of Parasitic Diseases, Wuxi, Jiangsu, 214064, People’s Republic of China; 2 Key Laboratory on Control Technology for Parasitic Diseases, Ministry of Health, Wuxi, Jiangsu, 214064, People’s Republic of China; 3 School of Public Health and Primary Care, the Chinese University of Hong Kong, Satin, Hong Kong; 4 National Institute of Parasitic Diseases, Chinese Center for Disease Control and Prevention, Shanghai, 200025, People’s Republic of China; 5 WHO Collaborating Center for Malaria, Schistosomiasis and Filariasis, Key Laboratory on Biology of Parasite and Vector, Ministry of Health, Shanghai, 200025, People’s Republic of China

**Keywords:** Schistosomiasis, Molluscicides, Cost-effectiveness, Control strategy

## Abstract

**Background:**

The application of chemical molluscicides is still one of the most effective measures for schistosomiasis control in P. R. China. By applying diverse molluscicide treatment scenarios on different snail densities in the field, we attempted to understand the cost-effectiveness of molluscicide application so as to prescribe an optimal management approach to control intermediate host snail *Oncomelania hupensis* under acceptable thresholds based on the goal of the National Schistosomiasis Control Programme.

**Methods:**

The molluscicidal field trial was carried out in the marshland of an island along the Yangtze River, Jiangsu province, P.R. China in October 2010. Three plots in the island representing low-density, medium-density and high-density groups were identified after the baseline survey on snail density. Each snail density plot was divided into four experimental units in which molluscicide (50% niclosamide ethanolamine salt wettable powder) was applied once, twice, trice and four times, respectively. The logistic regression model to correlate snail mortality rate with the covariates of number of molluscicidal treatment and snail density, and a linear regression model to investigate the relationship between cost-effectiveness and number of molluscicidal treatment as well as snail density were established.

**Results:**

The study revealed that increase in the number of molluscicide treatments led to increased snail mortality across all three population density groups. The most cost-effective regimen was seen in the high snail density group with a single molluscicide treatment. For both high and low density groups, the more times molluscicide were applied, the less cost-effectiveness was. However, for the median density group, the level of cost-effectiveness for two applications was slightly higher than that in one time.

**Conclusions:**

We concluded that different stages of the national schistosomiasis control/elimination programme, namely morbidity control, transmission control and transmission interruption, should utilize different molluscicide treatment strategies to maximize cost-effectiveness.

## Background

Schistosomiasis japonica is an infectious disease that severely threatens human health in the People’s Republic of China (P. R. China). It is one of the four major infectious diseases in alliance with AIDS, TB, and Hepatitis B, that the Chinese government has targeted for control during the period of the 11th and 12th Five-year Plan (2005–2015) [1]. The implementation of the National Schistosomiasis Control Program, with a new integrated strategy for schistosomiasis control recommended by MOH in P. R. China, has achieved significant success in the highly endemic areas [2]. The new strategy aims to interrupt the contamination of schistosome eggs in the environment with emphasis on fencing of water buffaloes (the major transmission reservoir), chemotherapy, access to clean water and adequate sanitation along with health education [3]. It was proposed that the achievement of the integrated strategy would be further strengthened by the snail elimination approach. As *Oncomelania hupensis* is the sole intermediate host snail of *Schistosoma japonicum*, extirpation of the snail makes it possible to cut off the transmission dynamics of schistosomiasis [4]. However, it is still not yet clear what is the best way to treat the snail in a cost-effective way due to the complex of various impact factors, including environmental, biological and social factors [4]. To date, application of chemical molluscicides is one of the most effective measures for snail control [5]. Niclosamide is now the only chemical molluscicide used in P. R. China as well as the only molluscicide recommended by World Health Organization (WHO) due to its high efficiency, low toxicity and that it causes comparatively little environmental contamination. To ensure its practical application, 50% niclosamide ethanolamine salt wettable powder (50% NESWP), which has higher dissolution, has been currently applied widely in the field of P. R. China [5-7].

Molluscicide treatment is always regarded as a time-consuming and lower cost-effective strategy in the schistosomiasis control programme, due to the following reasons. Firstly, large snail infested areas along the river and lake banks made the control activity a time-consuming and tough process [8], since it usually required two or three applications of molluscicidal agent i.e. using a spraying method, to reduce the snail density [9]. Secondly, molluscicidal intervention in the field and labour costs made the procedure expensive. Thirdly, the snail density fluctuated dramatically subsequent to the annual flooding [10-13]. It showed a high reproduction rate at low densities for population compensation [14,15].

Previous studies pertaining to the cost-effectiveness analysis (CEA) on the chemical molluscicidal treatment and optimizing the molluscicidal application strategy based on both the field experiments and modeling were rarely reported [3]. Hence, our ongoing work is focused on maximizing the efficiency of snail control strategies in order to further reduce disease transmission dynamics by lowering the intermediate host snail density. Using this approach, it is expected that molluscicidal intervention could add value into the new integrated strategy leading to elimination of schistosomiasis, and re-emergence of schistosomiasis could be contained post-elimination of schistosomiasis.

In this study, we aimed at assessing the cost-effectiveness of diverse molluscicidal control scenarios on different snail densities in the field to prescribe an optimal approach to control snail populations within one season treatment. The molluscicidal field trial was carried out in the marshland of an island along the Yangtze River, Jiangsu province, P.R. China.

## Methods

### Study site and study groups

In late October 2010 while the water level of the Yangtze River went down, the marshland along the river in Yangzhou, Jiangsu province was targeted as study field. A baseline survey on snail density [16] was carried out on a large island with identical ecological environment. Snails were collected and analyzed at 5-step intervals within the survey region. A square frame (0.11 m^2^) was placed at each collecting site. All adult snails within the frame were collected into envelopes and labeled with a location ID. The density of snails were calculated as - No. of live snails within the total number of frames / total number of frames.

Three plots in the island representing low-density group (< 3 snails / 0.1 m^2^), medium-density group (5–15 snails / 0.1 m^2^) and high-density group (> 15 snails / 0.1 m^2^) were identified after the baseline survey. All study plots were ecologically similar and the only factor differing between them was the number of molluscicidal treatments. Before snail density survey, tall reeds were cut and prepared for upcoming molluscicidal trials.

Each snail density plot was divided into four experimental units in which molluscicide was applied once, twice, trice and four times, respectively. To avoid the findings generated by chance, we quantitatively increased the study unit, which covered 100 m^2^ areas (10 m × 10m). Each unit was surrounded by stakes and wire mesh to prevent the migration of the snails. There were about 2–3 metres’ intervals between each unit. The non-experimental regions between and surrounding the study plots were also treated with molluscicide to avoid the possible migration snails. A control group was chosen in a separated open area with the similar ecological conditions as the treatment plots in the same island.

### Molluscicide treatment experiment

**Figure 1:**
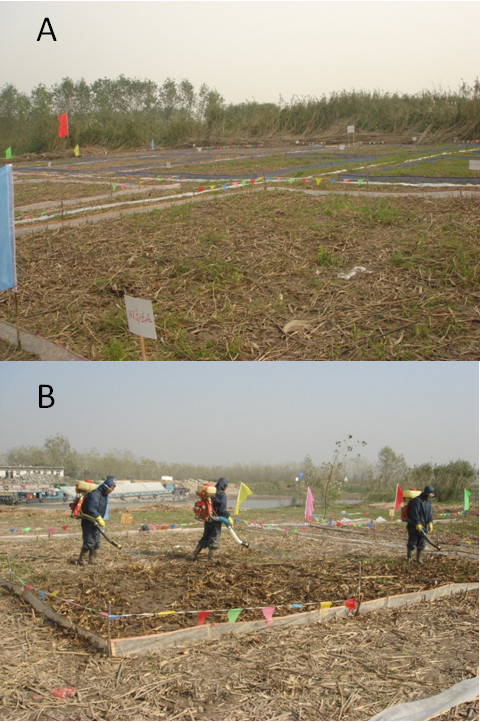
Study units (A) and molluscicide application by mist spraying machine (B).

Following the instruction of the schistosomiasis control manual [16], a total of 200g 50% NESWP were mixed with 100kg of water to make the molluscicidal liquid with a concentration of 0.2% [5]. By using a mist spraying machine (Figure 1), 100kg of molluscicidal liquid was evenly sprayed in one trial unit which was equivalent to the drug dosage of 2g/m^2^. A three day interval was required for the trial unit requiring more than 1 time of molluscicide treatment. It has been recorded that a 3 day interval between molluscicide treatments was long enough for the recovery of surviving snails [17]. Once all spraying activities finished, snail mortality rate was evaluated on D3, D7 and D15 post spraying, respectively. A total of 10 frames (0.1m^2^ per frame) in each research unit were investigated every time by random systematic sampling. All snails within the sampling frames were collected and the numbers of dead and alive snails were recorded.

### Cost-effectiveness assessment

Chinese Yuan (CNY) is the local currency of P. R. China (1USD = 6.3CNY). The cost of 50% NESWP is 34,400 CNY/ton. The recommended application dosage for molluscicidal agent was 2g/m^2^ in the marshland. Therefore, the cost of molluscicide once for each unit (100m^2^) was around 7 CNY.

Labor costs of each person was 80 CNY / day. Each person per day could spray 300 m^2^. Machinery depreciation was calculated by 0.05 CNY /m^2^. For each unit (100m^2^), the cost of the labor and machinery depreciation was about 31.7 CNY. The total cost of applying molluscicide in one unit (100m^2^) was 38.7 CNY.

$${\text{Mortality}}_{\text{Adjusted}}=\frac{{(\text{Mortality}}_{\text{Experiment}}{\;\text{-Mortality}}_{\text{Control}})}{{(1-\text{Mortality}}_{\text{Control}})}\times 100\%$$

Where Mortality_Adjusted_, Mortality_Experiment_ and Mortality_Control_ denote adjusted snail mortality, snail mortality in experimental group and snail mortality in control group, respectively.

$$\text{Cost}-\text{effectiveness}={\text{Mortality}}_{\text{Adjusted}}/\text{Total cost}$$

The logistic regression model was applied to snail mortality rate with the covariates of number of molluscicidal treatment and snail density.

$$\begin{array}{c} \text{logit}\left({\text{Mortality}}_{\text{Adjusted}}\right)\sim \text{No. spraying times}\\ +\text{Medium density}+\text{High density} \end{array}$$

A linear regression model was used to investigate the relationship between cost-effectiveness (log transferred) and number of molluscicidal treatment as well as snail density.

$$\begin{array}{c} \text{Log}\;\left(\text{Cost}-\text{effectiveness}\right)\sim \text{No. spraying times}\\ +\text{Medium density}+\text{High density} \end{array}$$

## Results

**Figure 2:**
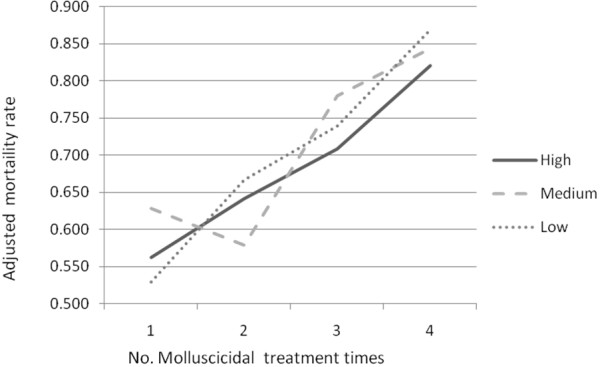
Adjusted snail mortality of molluscicidal spray in different density groups.

Within all three density groups, the adjusted mortality rates of snails were shown to increase with the molluscicidal spreading (Figure 2, Table 1).

**Table 1:** Molluscicide treatment impact in different snail density groups by 3 days, 7 days and 15 days post spraying

	**3 DAYS**				**7 DAYS**					**15 DAYS**			
**Spray times**	**Snail density group**	**Total No. Snails (10 frames)**	**No. Live snails (10 frames)**	**Mortality (%)**	**Adjusted mortality (%)**	**Total No. Snails (10 frames)**	**No. live snails (10 frames)**	**Mortality (%)**	**Adjusted Mortality (%)**	**Total No. Snails (10 frames)**	**No. live snails (10 frames)**	**Mortality (%)**	**Adjusted mortality (%)**
1	H	156	71	54.49	52.83	161	66	59.01	57.40	164	73	55.49	53.93
	M	61	19	68.85	67.72	73	35	52.05	50.17	76	26	65.79	64.59
	L	14	7	50.00	48.18	20	9	55.00	53.24	17	10	41.18	39.11
	C	57	55	3.51		53	51	3.77		59	57	3.39	
2	H	167	72	56.89	56.10	168	55	67.26	66.00	152	49	67.76	66.55
	M	45	19	57.78	57.01	70	28	60.00	58.46	57	26	54.39	52.66
	L	23	10	56.52	55.73	21	8	61.90	60.44	19	5	73.68	72.69
	C	56	55	1.79		54	52	3.70		55	53	3.64	
3	H	142	43	69.72	68.48	179	44	75.42	73.97	154	52	66.23	64.88
	M	53	10	81.13	80.36	63	11	82.54	81.51	63	18	71.43	70.29
	L	18	5	72.22	71.09	24	7	70.83	69.12	28	7	75.00	74.00
	C	51	49	3.92		54	51	5.56		52	50	3.85	
4	H	136	27	80.15	79.37	166	24	85.54	85.03	168	34	79.76	79.40
	M	57	7	87.72	87.24	66	8	87.88	87.45	70	17	75.71	75.28
	L	25	4	84.00	83.37	17	2	88.24	87.82	28	5	82.14	81.82
	C	53	51	3.77		58	56	3.45		57	56	1.75	

Note: H = high density group, M = median density group, L = low density group.

The logistic regression model also proved that the snail mortality rate was positively correlated with number of molluscicide treatment times with a coefficient of 0.415 (P<0.05, Table 2), although there was no significant difference between the density groups (P>0.05, Table 2).

**Table 2:** Summary of results of logistic and linear regression models

	**Coefficients**	**Estimate**	**Std. Error**	**z value**	**Pr(>|z|)**
Logistic regression model	Intercept	−0.128	0.292	−0.436	0.663
	No. of molluscicide application times	0.415	0.065	−6.376	**<0.001**
	Medium Density	0.036	0.285	0.127	0.899
	High Density	−0.101	0.262	−0.386	0.699
Linear regression model	Intercept	−4.339	0.134	−32.478	**<0.001**
	No. of molluscicide application times	0.415	0.042	−5.021	**0.001**
	Medium Density	−0.093	0.116	−0.802	0.445
	High Density	0.054	0.116	0.465	0.654

Note: *Statistical significant coefficients are shown in bold.

**Figure 3:**
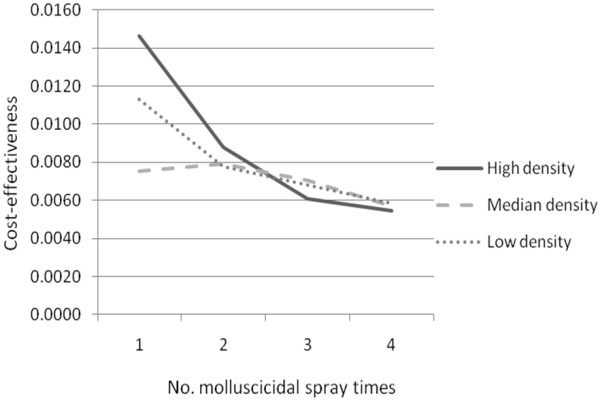
Cost-effectiveness comparison of molluscicidal spray in different density groups.

Results of cost-effectiveness analysis were presented in Figure 3. The most cost-effective regimen was seen in the high snail density group with a single molluscicide treatment. For both high and low density groups, the more times molluscicide was applied, the less cost-effectiveness it was. However, for the median density group, the level of cost-effectiveness for two applications was slightly higher than that in one time.

According to the results of the regression model for cost-effectiveness analysis, statistical significance existed between different times of molluscicidal treatment (P<0.05, Table 2). However, there was no difference between the density groups (P>0.05, Table 2).

## Discussion

Through the National Schistosomiasis Control Programme over the past 6 decades, P.R. China has been enormously successful in managing to reduce the extent of the endemic areas [10]. At present, transmission foci of schistosomiasis still remains in the middle and lower reaches of the Yangtze River valley in 5 provinces of Hunan, Hubei, Jiangxi, Anhui and Jiangsu and in some mountainous areas of Sichuan and Yunnan provinces [18]. By 2011, Hunan, Hubei, Jiangxi, and Anhui provinces reached the criteria of morbidity control, while Jiangsu as well as two mountainous provinces, namely Sichuan and Yunnan, have reached the criteria of transmission control and now are moving towards transmission interruption in another 5 years [19]. Comparing the reference criteria in different control stages, the requirements of remaining snail habitats were various (Table 3) [20]. Therefore, the control efforts on molluscicide treatment should be contrasted accordingly. It is necessary to carry out an economic analysis, such as CEA by comparing the relative costs and outcomes in terms of molluscicidal effects, to determine the best control strategy at different control stages [19].

**Table 3:** National criteria for control and elimination of schistosomiasis in P.R. China

**Stages**	**Criteria**
**Stage I.**	1. The infection rate in residents should be less than 5%.
Morbidity control of schistosomiasis	2. The infection rate in domestic animals should be less than 5%.
	3. No outbreak of acute schistosomiasis cases.
**Stage II.**	1. The infection rate in residents or domestic animals should be less than 1%.
Transmission control of schistosomiasis	2. No acute schistosomiasis case infected locality.
	3. No infected *Oncomelania* snail should be detected for at least 2 successive years.
	4. Data reflecting the changes in human and domestic animal infections and in snail examinations at administrative village level should be available.
**Stage III.**	1. No new infection should be found either in human or in domestic animals for 5 successive years.
Transmission interruption of schistosomiasis	2. No *Oncomelania* snail should be found after careful surveys for at least 2 years.
	3. Data and documents, and plans and measures for surveillance and consolidation of the control work should also be available.
**Stage IV.**	If no new infection in human and domestic animals has been detected for 5 successive years after reaching the criteria of transmission interruption, the area can be declared as an area of elimination for schistosomiasis, whereas consolidation and surveillance of the control work should be continued.
Elimination of schistosomiasis	

Currently, the schistosomiasis transmission status in P. R. China can be classified into four types, namely morbidity control, transmission control, transmission interruption, and elimination (Table 3), and the first three types of areas need to implement molluscicidal treatment in the control programme [18]. First, the transition stage from morbidity control to transmission control, such as Hunan, Hubei, Jiangxi and Anhui provinces, which are supposed to reach the “transmission control” by 2015. To achieve this goal of “No infected *Oncomelania* snail should be detected for at least 2 successive years”, the most important issue is to control the infection resources. There is no criterion pertaining to the snail density, but previous study detected the positive linkage between the positive snail distribution and snail density [21]. It is necessary to reduce the snail density to a certain level in the above mentioned four lake region provinces. Based on our study results, the molluscicidal effects positively related to the number of spraying times. However, the CEA indicated that the optimal spraying times should be one which maintains the snail population at a certain degree but does not increase the transmission risk significantly. Therefore, we recommend that the molluscicide treatment used once a year accompanied by the integrated control strategy with emphasis on the infectious source control was the best way in the pre-transmission control stage (Table 4).

**Table 4:** Recommended strategies in schistosomiasis control

**Control stages of schistosomiasis**	**Provinces**	**Recommended strategy**
Morbidity control	Hunan, Hubei, Jiangxi and Anhui	Molluscicide application once a year accompanied with the integrated control strategy
Transmission control	Jiangsu, Sichuan, Yunnan	Molluscicide treatment plus environmental management of snail habitats
Transmission interruption or post elimination	Fujian, Guangxi, Zhejiang provinces and Shanghai	Molluscicide three times treatment plus environmental management of snail habitats

Second, the transition stage from transmission control to transmission interruption, such as Jiangsu province and two high altitude provinces of Yunnan and Sichuan, it is required to achieve the goal of “transmission interruption” by 2015. There is supposedly, “No *Oncomelania* snail should be found after careful surveys for at least 2 years”. According to a previous study, *O. hupensis* follow a strong negative feedback mechanism, whereby extremely low-density snail habitats following snail control can in fact produce the largest subsequent pulses of snails, and thus may diminish the snail control efficiency [22]. Therefore, if the snail density post molluscicide treatment was over a certain threshold, the number of survival snails will exponentially grow. The study showed that there were still some remnant snails, although in small quantity, surviving even after 4 times’ molluscicidal application in the field. Elimination of snails for 2 successive years was an arduous task to fulfill. It is not an optimal process using molluscicides alone to reach the criteria of “transmission interruption” by 2015. Hence, we recommended that environmental modification of snail habitats could be an ultimate option (Table 4).

Third, during the stage of post-elimination/ transmission interruption, in regions such as Fujian, Guangxi, Zhejiang provinces and Shanghai municipality, the main task is sustainability and consolidating the obtained achievements. It is required not only to monitor the mobile population from transmission endemic regions, but also to suppress the snail density at very low levels. Once detected as a, re-emerging snail habitat, must be eliminated immediately taking less consideration of the cost, since the high cost could be compensation for the elimination of the disease (Table 4) [23].

The study was carried out in a closed quasi-field environment. It is assumed there was no snail migration during the experiment. However, the snail infested beaches along the Yangtze River is open. Especially, since the 1990s, the flooding frequency of the Yangtze River has been increased [24]. Affected by changes of water level with flooding, the migration of snails along the Yangtze River basin can change the population distribution significantly [23]. Therefore, the most difficult goal for the elimination of schistosomiasis in the marshland and lake region along the Yangtze River basin lies on the spread or/ and re-distribution of snails, which is the major driver for the resurgence of schistosomiasis. This is particularly applicable in the lower reaches of the Yangtze River region, where broad surface and slow velocity of the river provide favorable conditions for the islet formation and snail relocation [22]. Therefore, it is important to carry out quantitative research regarding the diffusion pattern and population dynamics of snails along the Yangtze River in combination with modern molecular approaches to trace the original distribution of the snail before migration, so as to effectively curb the re-emergence of the disease, as well as taking a more efficient surveillance and response strategy leading to the elimination of schistosomiasis in P.R. China.

In the study, the cost-effectiveness of diverse molluscicidal control scenarios on different snail densities was evaluated within one season treatment. It is warranted to carry on further study to maximize the efficacy of molluscicidal control throughout the whole year, e.g. Spring/ Autumn only vs multiple season combination, so as to establish the best regimen for seasonal molluscicidal control.

## Conclusions

The different stages of the National Schistosomiasis Control Programm in P.R.China, accompanied by the integrated control strategy with emphasis on the infectious source control, should utilize different molluscicide treatment strategies to maximize cost-effectiveness. It is recommended that, the molluscicide treatment used once a year from morbidity control to transmission control, environmental modification of snail habitats could be an ultimate option during the transition stage from transmission control to transmission interruption, and re-emerging snail habitats must be eliminated immediately during the stage of post-elimination/ transmission interruption.

## Competing interests

The authors declare that there are no competing interests.

## Author’ contributions

GJY and ZXN conceived the study and analyzed the data. GJY wrote the first version of the manuscript. XNZ revised the manuscript. All authors read and approved the final version of the manuscript. All authors read and approved the final manuscript.
